# Pulmonary arterial banding in mice may be a suitable model for studies on ventricular mechanics in pediatric pulmonary arterial hypertension

**DOI:** 10.1186/s12968-021-00759-8

**Published:** 2021-06-03

**Authors:** Melanie J. Dufva, Mario Boehm, Kenzo Ichimura, Uyen Truong, Xulei Qin, Jennifer Tabakh, Kendall S. Hunter, Dunbar Ivy, Edda Spiekerkoetter, Vitaly O. Kheyfets

**Affiliations:** 1 Department of Bioengineering, University of Colorado Denver, Denver, CO USA; 2 Department of Pediatrics, Section of Cardiology, Childrens Hospital Colorado, Aurora, CO USA; 3 Universities of Giessen and Marburg Lung Center (UGMLC), Justus-Liebig University Giessen, German Center for Lung Research (DZL), Giessen, Germany; 4 Department of Medicine, Division of Pulmonary and Critical Care Medicine, Stanford University, Stanford, CA USA; 5 Vera Moulton Wall Center for Pulmonary Vascular Disease, Stanford University, Stanford, CA USA; 6 Department of Pediatrics, Section of Cardiology, Children’s Hospital of Richmond, Virginia Commonwealth University, Richmond, VA USA; 7 Cardiovascular Institute, Stanford University, Stanford, CA USA; 8 Department of Bioengineering, University of Colorado Denver, 12700 E. 19th Ave, Aurora, CO 80045-2560 USA

**Keywords:** Pulmonary arterial hypertension, Pediatrics, Ventricular mechanics, FT-CMR, Tagged-CMR, Cardiovascular magnetic resonance

## Abstract

**Background:**

The role of interventricular mechanics in pediatric pulmonary arterial hypertension (PAH) and its relation to right ventricular (RV) dysfunction has been largely overlooked. Here, we characterize the impact of maintained pressure overload in the RV–pulmonary artery (PA) axis on myocardial strain and left ventricular (LV) mechanics in pediatric PAH patients in comparison to a preclinical PA-banding (PAB) mouse model. We hypothesize that the PAB mouse model mimics important aspects of interventricular mechanics of pediatric PAH and may be beneficial as a surrogate model for some longitudinal and interventional studies not possible in children.

**Methods:**

Balanced steady-state free precession (bSSFP) cardiovascular magnetic resonance (CMR) images of 18 PAH and 17 healthy (control) pediatric subjects were retrospectively analyzed using CMR feature-tracking (FT) software to compute measurements of myocardial strain. Furthermore, myocardial tagged-CMR images were also analyzed for each subject using harmonic phase flow analysis to derive LV torsion rate. Within 48 h of CMR, PAH patients underwent right heart catheterization (RHC) for measurement of PA/RV pressures, and to compute RV end-systolic elastance (RV_E_es_, a measure of load-independent contractility). Surgical PAB was performed on mice to induce RV pressure overload and myocardial remodeling. bSSFP-CMR, tagged CMR, and intra-cardiac catheterization were performed on 12 PAB and 9 control mice (Sham) 7 weeks after surgery with identical post-processing as in the aforementioned patient studies. RV_E_es_ was assessed via the single beat method.

**Results:**

LV torsion rate was significantly reduced under hypertensive conditions in both PAB mice (p = 0.004) and pediatric PAH patients (p < 0.001). This decrease in LV torsion rate correlated significantly with a decrease in RV_E_es_ in PAB (r = 0.91, p = 0.05) and PAH subjects (r = 0.51, p = 0.04). In order to compare combined metrics of LV torsion rate and strain parameters principal component analysis (PCA) was used. PCA revealed grouping of PAH patients with PAB mice and control subjects with Sham mice. Similar to LV torsion rate, LV global peak circumferential, radial, and longitudinal strain were significantly (p < 0.05) reduced under hypertensive conditions in both PAB mice and children with PAH.

**Conclusions:**

The PAB mouse model resembles PAH-associated myocardial mechanics and may provide a potential model to study mechanisms of RV/LV interdependency.

**Supplementary Information:**

The online version contains supplementary material available at 10.1186/s12968-021-00759-8.

## Introduction

Pulmonary arterial hypertension (PAH) is a condition characterized by an occlusive pulmonary vasculopathy that leads to increased afterload on the right ventricle (RV) and a progressive decline in ventricular function, which left untreated results in eventual RV failure and death [1, 2]. The RV and the left ventricle (LV) structurally share the interventricular septum and are, thus, undoubtedly a mechanically interdependent system [3]. Although historically PAH has been treated with RV function solely in mind [4], our recent research has shown altered LV mechanics with reduction in LV torsion and LV circumferential strain in pediatric PAH populations, which is associated with impaired RV contractility and function [5, 6]. Early research performed by Damiano et al*.* demonstrated that in electrically isolated canine hearts, approximately 70% of RV pressure and pulmonary flow was generated during LV contraction alone [7] emphasizing the importance of proper LV function for normal RV function. A study on patients with a systemic-loaded RV demonstrated that LV mechanical dysfunction in ventricular strain and twisting had a significant effect on RV mechanics [8], and others have shown prognostic value in reduced LV and RV myocardial strain for prediction of adverse outcome in both adults [9] and children [10]. These studies are strongly indicative of the LV’s influence on RV function, yet they do not investigate altered ventricular mechanical interdependence longitudinally in PAH, nor explore potential therapeutic interventions. Furthermore, there are distinct differences in the pathophysiology of PAH between adults and children—the latter showing increased vasoreactivity, a longer anticipated lifespan, and a higher association with congenital heart disease and developmental lung diseases [2, 11, 12], all factors that might influence ventricular mechanics. The mechanism of disease progression is likely different between children and adults [12]; hence, extrapolation of adult studies to the pediatric population is problematic and merits separate study.

A better understanding of the mechanism(s) of biventricular mechanics in PAH progression, its ability to influence RV function, and its potential therapeutic capacity, robust longitudinal animal studies are required. Animal models of pulmonary artery (PA) stenosis through application of a PA band provides a unique setting to study the effects of RV pressure overload that is consistent in severity and is a purely mechanical insult, which is free of injury to the pulmonary or systemic vasculature. Here we characterize the ventricular mechanics of a preclinical mouse model 7 weeks after surgical PA banding (PAB)—in which the RV is overloaded via mechanical constriction of the proximal PA—and compare myocardial biomechanics to human pediatric PAH subjects. The objective of this study was to investigate if the PAB mouse model emulates myocardial mechanics of children with PAH and thereby, may serve as a potential resource to study mechanisms of RV/LV interdependency in the context of the pressure-overloaded RV.

## Methods

To derive metrics of ventricular mechanics, we acquired balanced steady state free precession (bSSFP) and tissue-tagged cardiovascular magnetic resonance (CMR) images of healthy children, children with PAH, control mice (Sham) and PAB mice 7 weeks after surgery. We applied a feature-tracking (FT) algorithm (cvi42, Circle Cardiovascular Imaging, Calgary, Alberta, Canada) to analyze bSSFP CMR images for deriving myocardial strain and calculating LV torsion rate by Harmonic Phase Flow Analysis (Computer Vision Center, Universitat Autònoma de Barcelona, Barcelona, Spain [13, 14]).

### Study approval

All animal experiments were performed at Stanford University and in accordance with National Research Council guidelines (*Guide for Care and Use of Laboratory Animals*) and approved by local authorities (APLAC, Stanford University, Protocol #27626).

All human subject studies were performed with the approval of the Colorado Multi-Institutional Research Board (IRB) in accordance with the Declaration of Helsinki. All participants provided written and informed consent, prior to inclusion for a prior study. Parental consent was obtained for all subjects younger than 18 years. Informed consent for the current study was waived by the IRB.

### Mouse study protocol

Male C57Bl6 mice (10–14 weeks of age, corresponding to 21–23 human years) underwent either PAB around a 24G needle to induce moderate RV pressure overload or Sham surgery [15]. Only male mice were used to reduce the experimental variability. All mice pre-emptively received 0.05–0.1 mg/kg buprenorphine bodyweight subcutaneously along with constant isoflurane (2–3%) anesthesia during surgery. Only animals with a peak pressure gradient across the PAB > 20 mmHg (measured by echocardiography, Vivid 7, General Electric Healthcare, Waukesha, Wisconsin, USA) 1 week after surgery were included into the study protocol. Given these conditions, 7 weeks post-PAB surgery has been shown to induce significant RV dysfunction while maintaining adequate survival [15, 17]. At week 7 after surgery, all mice underwent small animal CMR and subsequent terminal intra-cardiac hemodynamic catheterization [16, 17]. Terminally after catheterization, all mice were killed by exsanguination. In total, 9 Sham and 13 PAB mice were originally included in this study. Of note, 1 PAB mouse died 4 weeks after surgery, leaving a total of 12 PAB mice in the study.

### Human subject population

Patients cared for in the Pulmonary Hypertension Program at Children’s Hospital Colorado, who had undergone clinically indicated right heart catheterization (RHC) and CMR within 48 h of each other, were retrospectively selected for this study. Control healthy subjects age 7–21 years with no known cardiopulmonary disease had been recruited via campus advertisement for a previous study [5] and were retrospectively selected to age-match PAH subjects to undergo CMR alone. The inclusion criteria for the PAH group were any person age 7–21 years, with mean pulmonary artery pressure (mPAP) ≥ 25 mmHg established by RHC or RV pressure ≥ 50% of systemic arterial pressure established by echocardiogram before age 18 years for PAH patients. We excluded significant intracardiac shunts defined as a pulmonary: aortic flow of more than 1.2:1. Furthermore, patients with pulmonary thromboembolic diseases, PAH from left heart disease, veno-occlusive disease, pulmonary capillary hemangiomatosis, or lung disease were excluded from this study. Medical records, including World Health Organization (WHO) functional class, were retrospectively reviewed.

For any subject, differences in hemodynamic states between sedated RHC and non-sedated CMR were unavoidable. To minimize these differences, RHC and CMR were performed on the same day whenever possible for each patient. Otherwise, the procedures were done within a maximum 48 h of each other and was enforced as inclusion criterion for the study. Our institutional protocol calls for RHC under general anesthesia for all children, while our CMR research protocol included older children (> 7 years old) without sedation during CMR. Therefore, with a minimum subject age of 7 years for this study, no children underwent sedation during CMR.

### Hemodynamic parameters measured in children

Pulmonary vascular resistance (PVR) was defined as a measure of resistive afterload [PVR = (mPAP − PCWP)/CO)], where mPAP is mean pulmonary arterial pressure, PCWP is the pulmonary capillary wedge pressure, and CO is cardiac output. Cardiac index was measured using the Fick’s principle at baseline condition, with FiO_2_ as close to room air as tolerated.

#### RV end systolic elastance

For assessment of RV contractility, RV end-systolic elastance (E_es_) was calculated using the single beat method described by Truong et al. [18, 19]. RV pressure waveforms generated from RHC were analyzed using the single beat method for both humans and mice. E_es_ was estimated as the ratio of the difference between “maximum theoretical pressure” and end systolic pressure to stroke volume [(P_max_ – P_es_)/SV] using the Takeuchi method [20]. Arterial compliance was defined the ratio of stroke volume (SV) to pulse pressure (PP) [SV/PP]. Arterial elastance (E_a_) (inverse of arterial compliance) was defined as the ratio of end systolic pressure to stroke volume [P_es_/SV].

### Imaging parameters computed in children

CMR was performed on a 1.5T CMR scanner (Ingenia, Philips Healthcare, Best, the Netherlands) with dedicated cardiac receiver coils appropriate for pediatric subjects. All children were awake during the study. Retrospective-gated cine imaging was performed using breath-holds during expiration for all patients. bSSFP images were acquired at baseline, with parameters of TR 2.8–3.5 ms, echo time of 1.2–1.5 ms, 25 phases, 4–10 mm slice thickness, and acquired matrix size = 192 × 192. These parameters were selected based on subject size, and temporal resolution varied slightly based on subject heart rate. Tagged CMR images were acquired through application of spatially modulated magnetization (SPAMM) to nullify signal within defined parallel lines or grids that persist through the cardiac cycle. Tag persistence throughout the entire cardiac cycle was optimized by minimizing flip angle and utilizing binomial radiofrequency pulses. Typical tagged-CMR image parameters for human subjects were TR 3.9–4.7 ms, echo time of 3.7 ms, voxel size of 1.875 × 1.875 × 8 mm, 6–8 mm slice thickness, flip angle 15°, matrix 144 × 256, with a tag spacing of 6–8 mm. Images were acquired with a minimum of 25 phases throughout the cardiac cycle in short axis view at the basal, mid, and apical levels. The short-axis basal slice was demarcated by a full circumferential view of the LV chamber as landmarked directly inferior to the mitral valve leaflet tips. The mid-chamber slice was demarcated by the region of the cavity encompassing the entire length of the papillary muscles. The short-axis apical slice was demarcated by a full circumferential view of the chamber inferior to the papillary muscles but superior to the end of the cavity.

### Imaging parameters computed in mice

Small animal CMR was performed on a 7T scanner (BioSpec, Bruker Corp, Billerica, Massachusetts, USA) with all mice under isoflurane anesthesia. After induction of anesthesia, CMR-designated electrocardiogram (ECG) electrodes were attached to their paws for retrospective ECG gating, and mice were placed on a dedicated mouse platform inside the coil. Rodents then underwent bSSFP image acquisition and subsequent SPAMM-tagged CMR with tagging persistence optimized similarly for pediatric patients. bSSFP image parameters for mice were TR 6 ms, echo time of 2.5 ms, 1 mm slice thickness, acquired matrix size = 192 × 192, with 20 phases. Tagged CMR imaging parameters for mice were 1 mm slice thickness, TR 10 ms, echo time of 3 ms, flip angle 15°, matrix 128 × 256, voxel size of 0.15 × 0.15 × 1 mm, with a tag spacing of 0.6–0.8 mm. Tagged CMR images were acquired in LV short axis view at the basal, mid, and apical levels with 20 phases throughout the cardiac cycle. These parameters were chosen to ensure consistency of spatial sampling and standardization of the FT method used between mice and human subjects.

### Myocardial mechanics

#### Myocardial strain

**Fig. 1:**
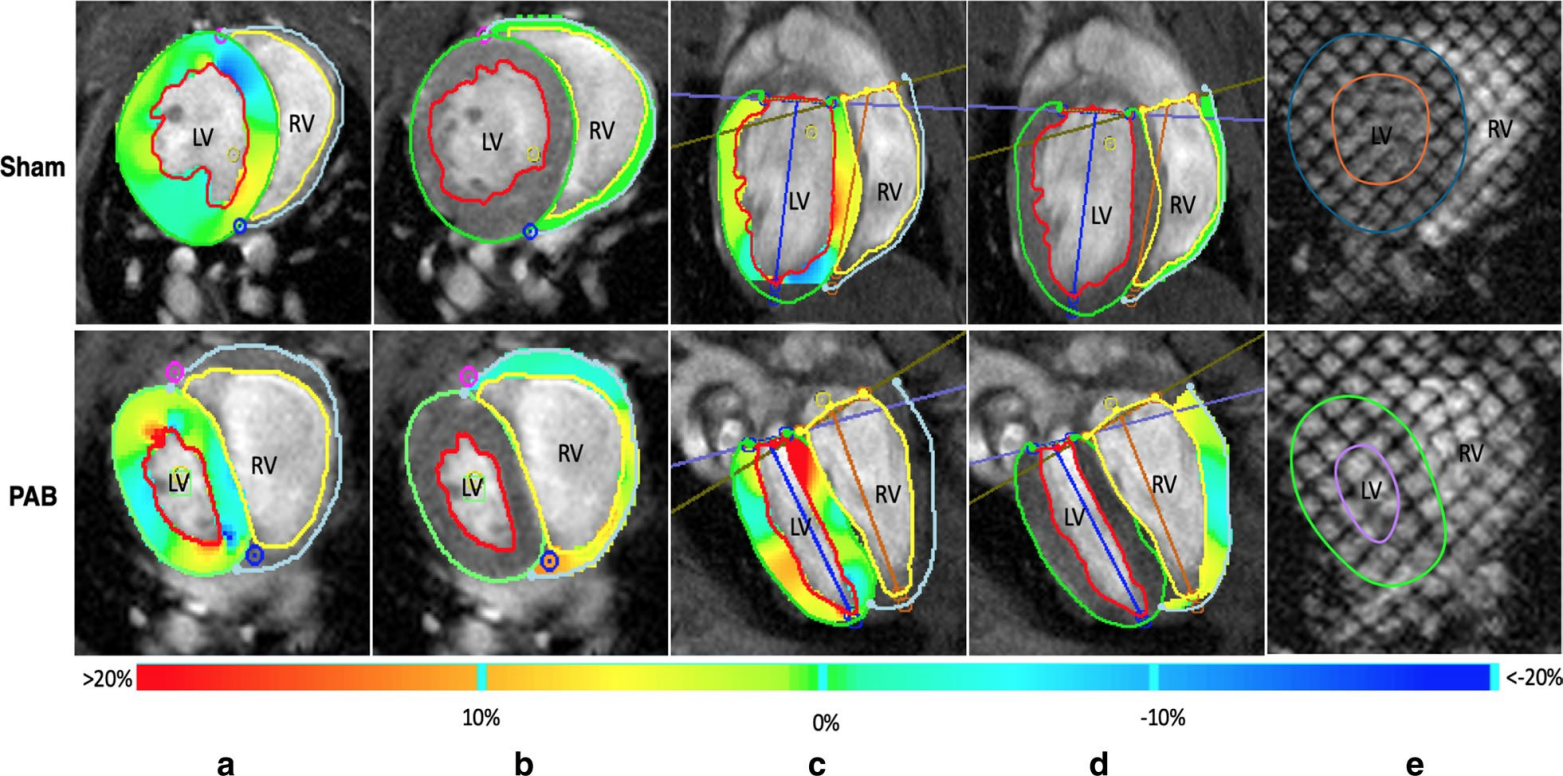
Balanced steady state free precession (bSSFP) and tagged cardiovascular magnetic resonance (CMR) images of a Sham (top) and pulmonary artery banded (PAB) (bottom) mouse used for derivation of myocardial strain and left ventricular (LV) torsion rate. bSFFP-CMR images showing CMR-feature tracking (FT) derived peak circumferential strain in short-axis view at end-diastole for the **a** LV and **b** the right ventricle; in long-axis view at end-diastole for the **c** LV and **d** the RV; **e** tagged-CMR with endo- and epi-cardial tracings of the LV at end-diastole

Myocardial strain is a measurement of mechanical deformation—either lengthening or shortening—of myocardial tissue [21]. Myocardial strain was calculated using feature-tracking software (cvi42; Circle Cardiovascular Imaging) from short-axis and horizontal long-axis bSSFP cine images. LV and RV endocardial and epicardial borders were manually traced at end-diastole in both short axis and horizontal long axis planes, with reference points marked for RV insertion points and mitral annular plane. LV epicardial tracings at the anteroseptal wall shared a mutual border with the endocardial tracings of the RV lateral wall. All LV strain analysis included the septum. Automatic feature tracking was performed on RV and LV myocardium for calculation of global radial, circumferential, and longitudinal peak strain (Fig. 1a–d). Directional strains were defined as follows: radial direction is normal to the mid-curve pointing outside the myocardium; circumferential direction runs along the circumference of the myocardium in the short axis plane, defined in such a way that the radial–longitudinal–circumferential coordinate system is right-handed; and longitudinal direction runs from the apex to the base [9, 21]. Peak strains and displacements, taken as the maximum absolute value, for each direction and for each ventricle were measured in all subjects and mice. This analysis was repeated three times and strain values were averaged. Intra- and interobserver variability was assessed for repeatability of the feature-tracking algorithm.

#### LV torsion rate

LV torsion rate describes the rate of twisting of the ventricle during systole or untwisting during relaxation in diastole [22]. LV torsion rate was calculated throughout the systolic cardiac cycle using Harmonic Phase Flow Analysis (Computer Vision Center, Universitat Autònoma de Barcelona), [13, 14]) from tagged CMR images. This method has been validated previously by Dufva et al. in a study of LV torsion in children with PAH [5]. Briefly, LV rotation in degrees was calculated over systole for basal, mid-ventricular, and apical short axis views. End systole was defined by aortic valve closure. Torsion was calculated using the equation T = (BR − AR)(R_apex_ + R_base_)/2D, where BR is the rotation of the base, AR is the rotation of the apex, R_apex_ and R_base_ are their respective radii, and D is the length between the base and apex in systole [23]. LV radius was defined as half the length of the measured distance between the inferior endocardial wall and the anterior endocardial wall (Fig. 1e). Torsion rates were calculated as the change in degrees of torsion over one systolic cycle and are reported as °/τ where τ is the period of % systole, also referred to as torsional shear angle [22]. This was done to normalize to the systolic phase of the cardiac cycle in order to remove the dimension of time and compare torsion rate between species of drastically different heart rates. Values reported are taken as the maximum LV torsion rate during systole ($$\frac{\partial T}{\partial \tau }{\left.\right|}_{max during systole}$$). Cubic spline interpolation was performed on torsion versus percent systole with an interval of 0.001. Planar vector fields were generated for each cardiac frame during systole. These vectors are then used to derive apical and basal rotation that are used to calculate torsion.

### Statistical analysis

All statistical analyses were performed in MatLab (Mathworks, Natick, Massachusetts, USA) and utilized an alpha of 0.05 for significance, with a 95% confidence interval. Mean and standard deviation were calculated—for control subjects, PAH patients, Sham, and PAB—for metrics of LV torsion rate and global radial, circumferential, and longitudinal peak strain. Metrics derived were tested for normal distribution using the Lilliefors test. Unless otherwise stated, values reported were determined to be normally distributed. An unpaired, two-tailed Student t-test was used to compare mean values of these metrics between control and PAH subjects and between Sham and PAB. To compare non-normally distributed variables we performed the Mann–Whitney test. ANOVA analysis was performed to compare means of LV torsion rate, global radial, circumferential, and longitudinal peak strain between the four groups. Univariate linear regression was used to assess relationships between LV torsion rate and RV contractility for PAH subjects and PAB.

Principle component analysis (PCA) was performed on the combined dataset of z-score normalized myocardial mechanics to determine if segregation between PH groups (PAH patients and PAB mice) and control groups (control subjects and Sham mice) was seen after a transformation along the first three principal components. Results are shown using biplots with a vector for each variable superimposed.

Intra-, inter-observer agreement and reproducibility for harmonic phase flow analysis—and the processing algorithm used—have been verified by us in a previous study [5]. Intraobserver and interobserver reproducibility of the feature-tracking algorithm was assessed utilizing intraclass correlation coefficients (ICC) and Bland–Altman analysis in 8 mice (4 Sham, 4 PAB) by two blinded researchers (J.T. and M.D.). Intraobserver variability was evaluated by comparing measurements performed by the same observer (M.D.) approximately 2 weeks apart in 8 mice (4 Sham, 4 PAB).

## Results

### Patient demographics

Demographics of the PAH pediatric sample are summarized in Table 1. All participants/guardians provided written and informed consent. Our study included 17 control and 18 PAH. Of note, all PAH patients were classified in WHO group I at the time of CMR/RHC. One patient was diagnosed as PAH due to a connective tissue disease, one patient had hereditary PAH, three had PAH due to congenital heart disease (two patients with atrial septal defects, one patient with a patent ductus arteriosus), and 13 of the PAH patients were diagnosed as idiopathic PAH. 66% of the PAH group and 65% of the control group were female. There was no significant difference in age between PAH and controls, with a median age of 13 (range 9–20) years for the PAH group and 10 (range 7–16) years for controls. RV ejection fraction (RVEF) was significantly lowered in PAH patients compared to control subjects. There were no significant differences observed between PAH and control groups for RV CO and SV, nor for their indexed values. Furthermore, the Lilliefors test revealed non-normal distribution for RV CO in PAH patients. Median mPAP for PAH subjects was 49 (range 24–58) mmHg and median PVR was 8.5 (range 2.3–22.8) Wood units. No significant difference in LV ejection fraction (LVEF) was observed between control subjects (median 59%, range 53–66%) and PAH patients (median 57%, range 42–65%).

**Table 1:** Patient demographics and hemodynamic characteristics

Parameter	PAH (n = 18)	Control (n = 17)	p-value
Female, n (%)	12 (66%)	11 (65%)	0.93
Age, y	13 (9–20)	10 (7–16)	0.08
Body surface area, m^2^	1.53 (0.83–1.76)	1.43 (0.98–1.77)	0.83
Height, cm	153.8 (123.7–163.1)	150.2 (127.4–176.1)	0.52
Weight, kg	50.8 (20.6–67.8)	46.6 (26.4–90.6)	0.98
WHO class I	18 (100%)	…	…
PVR (Pa s/m^3^)	8.5 (2.3–22.8)	…	…
mPAP, mmHg	49 (24–58)	…	…
PCWP (mmHg)	9 (5–12)	…	…
LVEF (%)	57 (42–65)	59 (53–66)	0.32
RVEF (%)	45 (22–54)	58 (47–67)	**< 0.001**
RV CO (mL/min)	5.6 (2.2–11.3)^a^	4.5 (2.5–8.4)	0.96
RV SV (mL)	76.2 (26.7–106.8)	63.4 (35.8–97.1)	0.15

Data are expressed as median values (interquartile range), n (%), unless otherwise noted

Bold p-values represent significance

*PAH* pulmonary artery hypertension, *WHO* World Health Organization, *PVR* pulmonary vascular resistance, *mPAP* mean pulmonary arterial pressure, *PCWP* pulmonary capillary wedge pressure, *LVEF* left ventricular ejection fraction, *RV* right ventricle, *RVEF* right ventricular ejection fraction, *CO* cardiac output, *SV* stroke volume

^a^Indicates non-normally distributed values tested with the Mann–Whitney test

### RV function

**Fig. 2:**
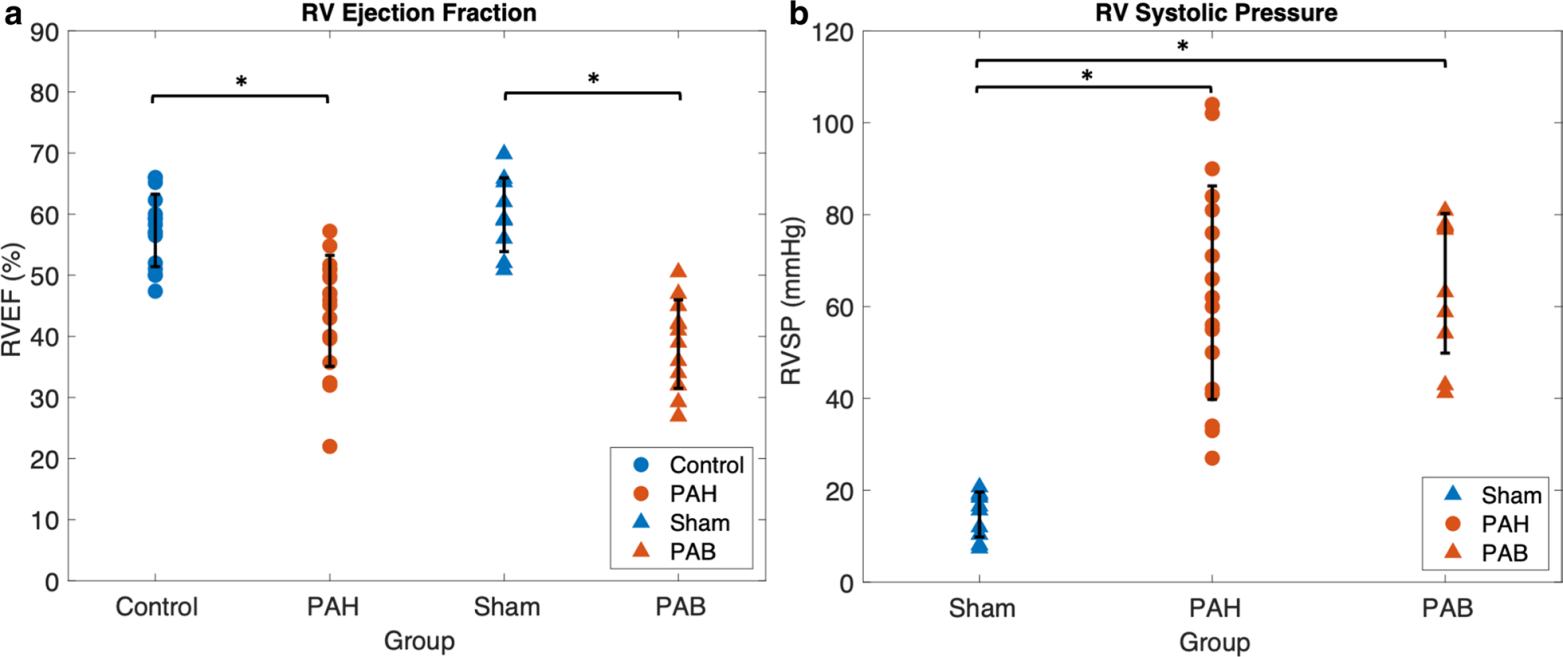
RV ejection fraction (RVEF) and RV systolic pressure (RVSP) are comparable between pulmonary artery hypertension (PAH) patients and PAB mice. **a** RVEF is reduced in PAH subjects (57 ± 6% in PAH versus 44 ± 9% in controls, p < 0.001) and PAB mice (62 ± 7% in PAB versus 37 ± 11% in Sham, p < 0.001) compared to control (CTRL) subjects and Sham mice, **b** RVSP is significantly increased in both PAH patients (63 ± 23 mmHg, p < 0.001) versus known healthy values [55], and PAB mice (64 ± 16 mmHg, p < 0.001) relative to Sham mice (19 ± 1 mmHg). Note, RVSP was not measured for control subjects. Variables were compared between control and hypertensive groups using a Student’s unpaired t-test with an α = 0.05. Data is expressed as mean ± SD. n = 18 in PAH cohort; n = 17 in control cohort; n = 12 in PAB group; n = 9 in Sham group

RVEF is shown in Fig. 2a for control subjects, PAH patients, Sham, and PAB. Mean RVEF was significantly decreased in PAH patients compared to control subjects (p < 0.001) and Sham (p < 0.001), respectively. There was no significant difference in RVEF between human control subjects and Sham (p = 0.75), or between PAH patients and PAB (p = 0.67). RV systolic pressure is shown in Fig. 2b for PAH patients, PAB, and Sham. RV systolic pressure was much higher in PAH patients (63 ± 23 mmHg) and similar to the RV systolic pressure after PAB in mice (64 ± 17 mmHg), compared to Sham (19 ± 1 mmHg) with no significant difference in RV systolic pressure between PAH patients and PAB mice (p = 0.84). Because control subjects did not undergo RHC, RV systolic pressure was not measured; however, RV size and function were assessed via CMR to verify phenotypic normal RV function.

### LV torsion rate and RV function

**Fig. 3:**
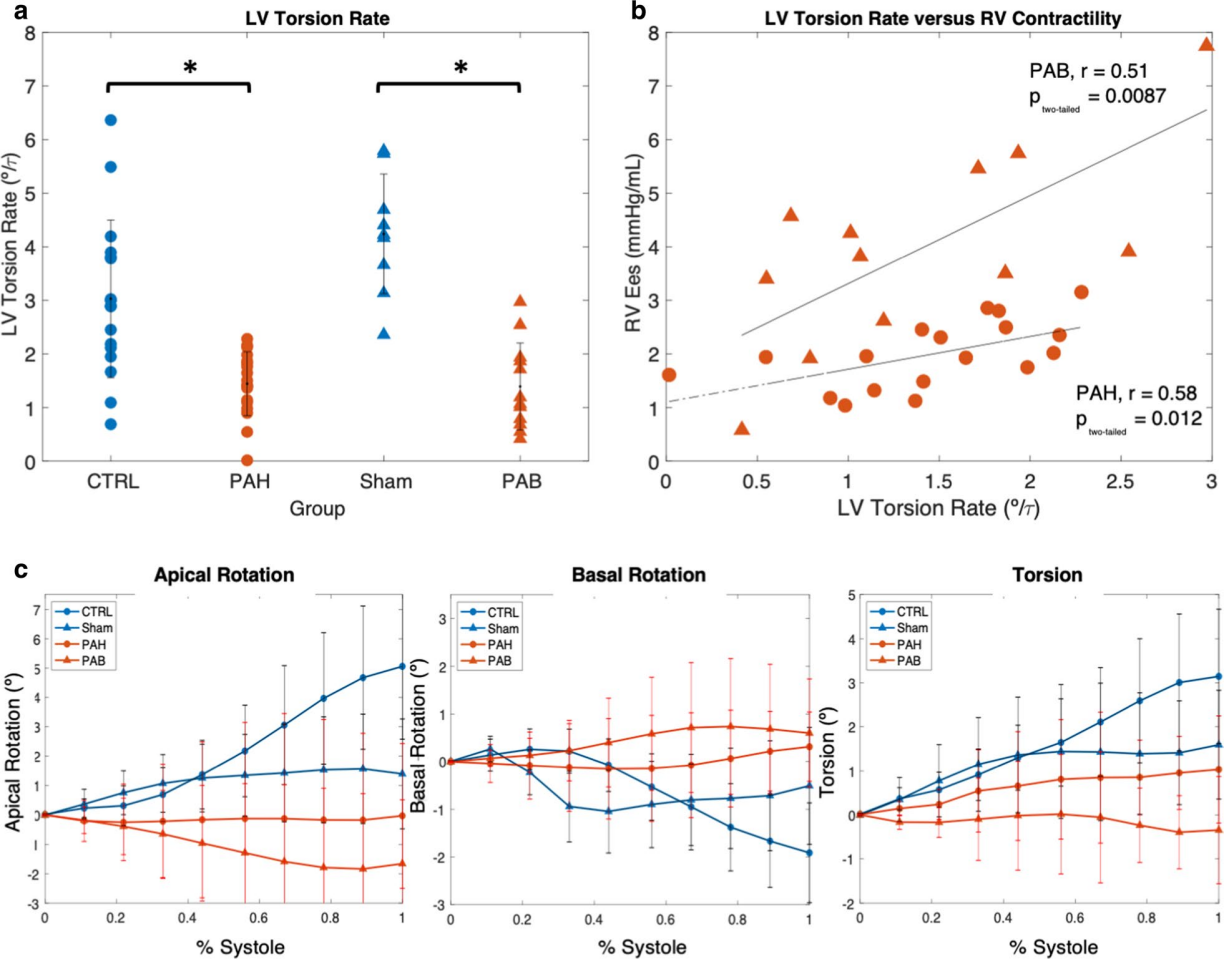
LV torsional mechanics are significantly different between Sham mice and PAB mice and comparable to human subjects. **a** LV torsion rate is reduced in PAH subjects (1.4 ± 0.6°/τ) and PAB mice (0.8 ± 0.6°/τ) compared to CTRL subjects (3.0 ± 1.5°/τ) and Sham mice (4.2 ± 1.4°/τ) (p < 0.001, p = 0.004, respectively) where τ is the period of % systole, **b** LV torsion rate is associated with RV contractility in PAB mice (▲, r = 0.91, p < 0.05) and strongly associated in PAH subjects (●, r = 0.51, p < 0.04), **c** Apical rotation, basal rotation, torsion are shown for control subjects (●, blue), PAH subjects (●, red), Sham mice (▲, blue), and PAB mice (▲, red) with error bars at intervals of 10%. Variables were compared between control and hypertensive groups using a Student’s unpaired t-test with an α = 0.05. Correlation coefficients were derived using univariate linear regression with an α = 0.05. Data are expressed as mean ± SD. n = 18 in PAH cohort; n = 17 in control cohort; n = 12 in PAB group; n = 9 in Sham group

Mean LV torsion rate and standard deviation for each group are shown in Fig. 3a. Mean LV torsion rate was significantly reduced for PAH subjects (1.4 ± 0.6°/τ) and for PAB (0.84 ± 0.6°/τ) compared to their control counterparts (3.0 ± 1.5°/τ, p < 0.001, and 4.2 ± 1.4°/τ, p = 0.004, control subjects and Sham, respectively). LV torsion rate correlated with RV contractility (measured as RV_E_es_ by single beat) for both PAH (r = 0.91, p = 0.05) patients and PAB (r = 0.51, p = 0.04). LV apical rotation, basal rotation, and torsion are shown in Fig. 3c for PAH patients (●, red), PAB (▲, red) mice, control subjects (●, black), and Sham (▲, black) mice with means and standard deviation presented at 11, 22, 33, 44, 56, 67, 78, 89, and 100% systole. The greatest differences were observed at end-systole for both rotation and torsion.

### Myocardial strain

The intraobserver study results for CMR-FT derived myocardial strain in 8 mice yielded good agreement with analysis performed 2 weeks apart (ICC, 0.92; mean difference, 0.85; 95% confidence interval − 0.10 to 1.8). Similarly, interobserver study results revealed good agreement as well (ICC, 0.85; mean difference, 1.1; 95% confidence interval − 0.12 to 2.28). These results are shown in Additional file 1: Figure S1.

**Fig. 4:**
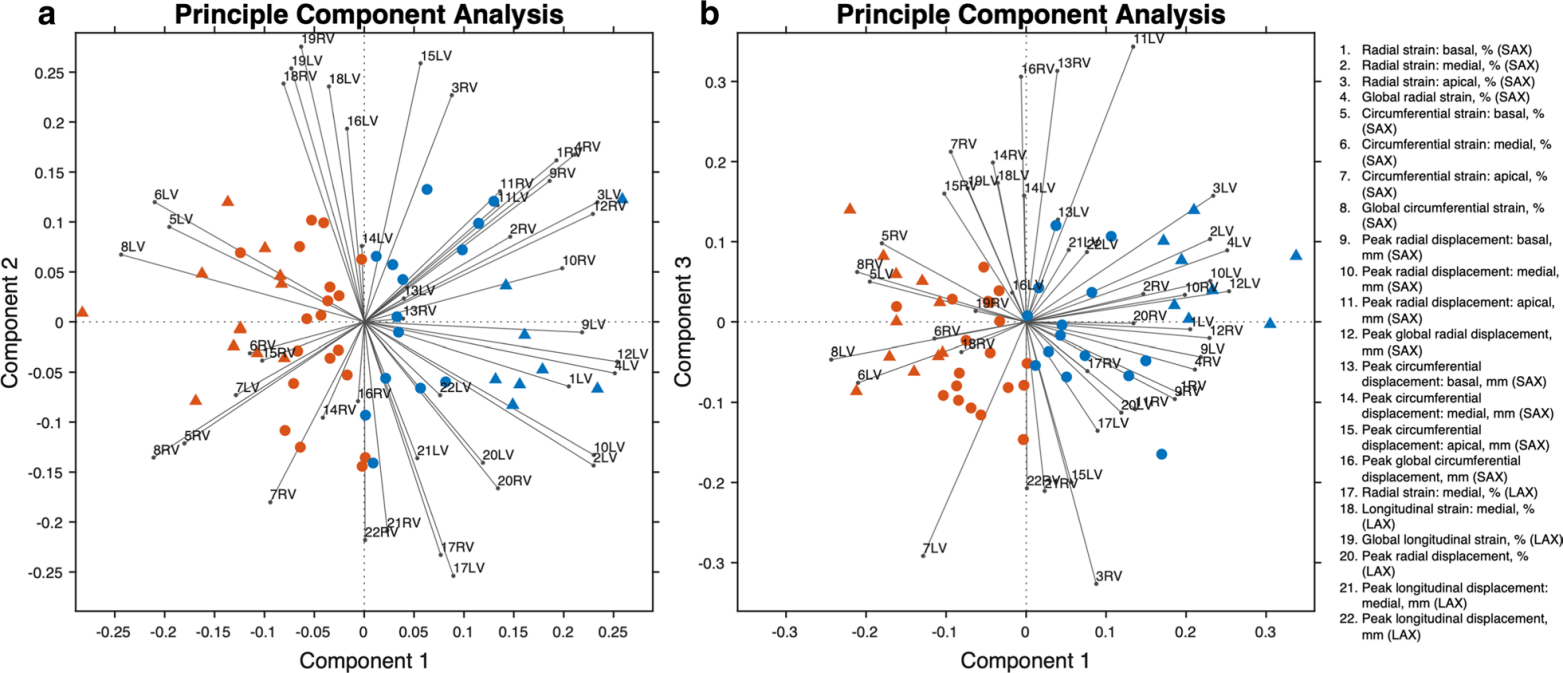
Principle component analysis (PCA) of 22 metrics of myocardial strain reveals distinct clusters based on disease status, with PAB mice (▲, red), Sham mice (▲, blue), PAH patients (●, red), and control subjects (●, blue); **a** PC1 versus PC2, and **b** PC1 versus PC3. n = 18 in PAH cohort; n = 17 in control cohort; n = 12 in PAB group; n = 9 in Sham group. The variable weights correspond to the strain parameters listed on the right

PCA was performed on the combined dataset of myocardial strains to further determine if grouping occurred between PAB mice and PAH patients, and between Sham mice and control subjects (see Fig. 4). Hypertensive (PAH patients and PAB mice) and normotensive (control subjects and Sham mice) groups revealed distinct clusters along PC1. Within each cluster, human subjects and rodents formed sub-clusters, but each subject was more distinctly clustered based on myocardial mechanics rather than species.

**Fig. 5:**
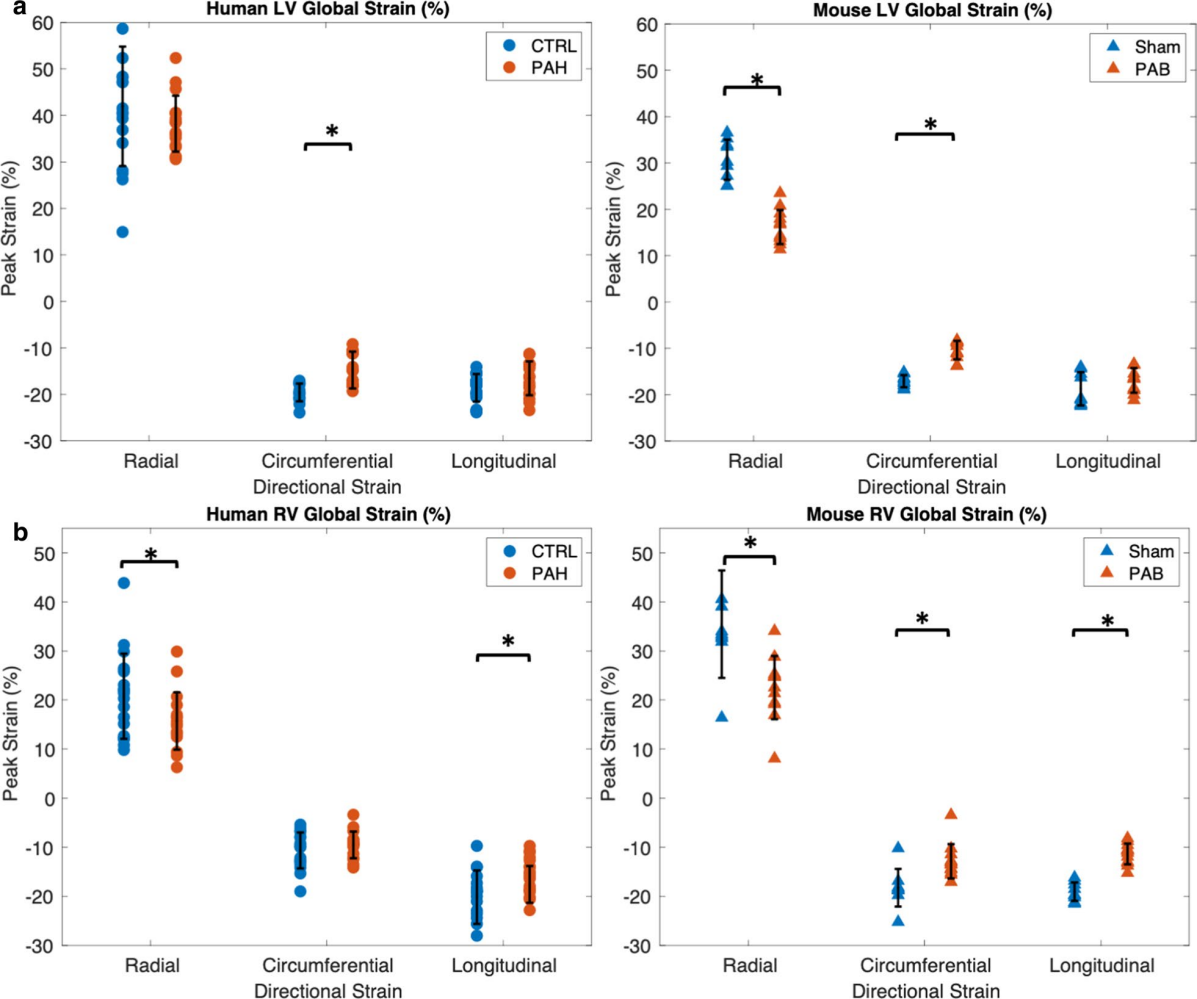
RV and LV global peak strains are significantly reduced in PAH subjects and PAB mice. **a** LV peak radial (26 ± 23% versus 33.9 ± 2%, p = 0.002) and circumferential (− 16 ± 1% versus − 18 ± 1%, p < 0.04) strain are reduced in PAB compared to Sham mice, while in children just LV peak circumferential strain (− 15 ± 4% versus -20 ± 2%, p < 0.02) is reduced in PAH compared to CTRL subjects. **b** RV peak radial (25 ± 3% versus 34 ± 3%, p = 0.002), circumferential (− 16 ± 1% versus − 19 ± 1%, p < 0.001) and longitudinal (− 18 ± 1% versus − 20 ± 1%, p = 0.02) strain are reduced in PAB compared to Sham mice, while in children peak radial (16 ± 5% versus 21 ± 8%, p < 0.05) and longitudinal (− 18 ± 4% versus − 20 ± 5%, p < 0.01) are reduced in PAH compared to CTRL subjects. Strain variables were compared between control and hypertensive groups using a Student’s unpaired t-test with an α = 0.05. Data are expressed as mean ± SD. n = 18 in PAH cohort; n = 17 in control cohort; n = 12 in PAB group; n = 9 in Sham group

Global peak radial, circumferential, and longitudinal strains for each cohort are shown in Fig. 5. Means and standard deviation for each parameter are shown in Table 2. Peak LV circumferential strain was significantly reduced in both PAH patients and PAB compared to their control counterparts. Peak LV radial strain was reduced in PAB compared to Sham. However, this reduction was not seen in human subjects, most likely due to the higher variability in human subjects. Peak RV radial and longitudinal strain were significantly decreased in both PAH patients and PAB compared to control subjects and Sham, respectively. Peak RV circumferential strain was reduced in PAB compared to Sham. However, this reduction was not significant in human subjects, again potentially due to the higher variability in human subjects.

**Table 2:** Global peak myocardial strain parameters

Global strain parameter	Control (n = 17)	PAH (n = 18)	p-value	Sham (n = 9)	PAB (n = 12)	p-value
LV peak radial strain (%)	42 ± 13	38 ± 6	0.28	34 ± 2	26 ± 3	**0.002**
LV peak circumferential strain (%)	− 20 ± 2	− 15 ± 4	**0.02**	− 18 ± 1	− 16 ± 2	**0.037**
LV peak longitudinal strain (%)	− 19 ± 3	− 17 ± 4	0.08	− 19 ± 4	− 17 ± 2	0.37
RV peak radial strain (%)	21 ± 8	16 ± 5	**0.04**	34 ± 3	25 ± 4	**0.002**
RV peak circumferential strain (%)	− 11 ± 4	− 10 ± 3	0.31	− 19 ± 1	− 16 ± 1.2	**0.001**
RV peak longitudinal strain (%)	− 20 ± 5	− 18 ± 4	**0.009**	− 20 ± 1	− 18 ± 2	**0.02**

Data are expressed as mean ± standard deviation. All values are in units of percent

Bold p-values represent significance.

## Discussion

In this pilot study we characterized and compared key indices of LV and RV mechanics by CMR imaging in pediatric PAH patients and a cohort of PAB mice. We demonstrate that (1) a decrease in LV torsion rate in PAH patients is also observed in RV pressure-overload in mice with a decreased RV contractility in both species; and (2) the response in global strain metrics to RV pressure overload are largely consistent between species. To our knowledge, this is the first characterization and comparison of myocardial mechanics in a pediatric PAH population with a preclinical rodent model of RV pressure overload via tagged-CMR and CMR-FT. These data suggest that PA-banded mice share the ventricular mechanics observed in the pediatric PAH population and that the PAB mouse model, therefore, could be beneficial for future mechanistic studies of inter-ventricular dependency that relate to pediatric PAH.

### RV functional metrics

To assess RV function, RVEF and RV systolic pressure were analyzed between groups. RVEF is an important prognostic metric for survival in PAH and is often used as a metric of RV function [1, 23], which has been shown to be a predictor of long-term outcome in children with PH with values of RVEF < 50% considered indicative of RV dysfunction [24]. RVEF was significantly decreased in both the PAH patients and PAB mice, compared to control subjects and Sham. Control and Sham subjects had comparable RVEF, in line with data from previous research [25]. RVSP was also significantly increased and similar in PAH patients and PAB mice compared to Sham. Depending on the severity of the PAB and, therefore, the degree of the pressure gradient across the pulmonary valve, the PAB model can be used to study RV hypertrophy with preserved overall RV function as well as with RV failure [26, 27]. While our data confirms a mildly reduced RV function, our moderate PAB mice did not have overt RV failure. In the pediatric PAH population, all subjects were categorized in WHO functional class (WHO-FC) I, which is considered the least symptomatic of PAH patients [28]. Furthermore, while RVEF was reduced, RV CO and RV SV were not, indicating a mild form of PH where CO is still maintained. Thus, upon preliminary comparison of RV function, the PAB mouse model may have the potential to serve as a surrogate model to the pediatric PAH population presented in this study.

### LV torsion rate

Damiano et al [7] along with other studies [29, 30] have all shown that mechanical energy transferred from LV contraction generates the majority of the pressure in the RV and pulmonary flow. Therefore, reduced LV torsion rate in both PAB mice and PAH subjects is intriguing, considering decreased RV contractility was also observed in both species. Although the current analysis does not demonstrate that decreased LV torsion rate as a causal mechanism for decreased RV contractility, which is likely multifactorial, it does warrant further investigation.

Multiple studies show that RV free wall remodeling -commonly seen under pressure overload—can mechanically decrease LV torsion [31, 43]. It has also been demonstrated that this decrease in LV torsion and torsion rate occurs within the first week of PAB in mice [31], which would further suggest that the effect is biomechanical stimuli from the remodeling and pressure overloaded RV free wall or septum. However, to our knowledge, there is no direct evidence to mechanistically link decreased LV torsion rate to decreased RV contractility in PAH. This is a topic of ongoing study, but there is implicit evidence to support this idea. LV torsion is known to play a major role in potential energy storage at end systole for diastolic recoil [32] and, as previously mentioned, the RV relies on mechanical energy transfer during systole for pressure generation [7]. However, how this relationship is impacted by RV pressure overload or the resulting remodeling remains unknown.

Of note, the reported units of LV torsion rate (°/τ) were normalized throughout the systolic phase of the cardiac cycle, rather than in the time domain, in order to compare values of vastly different heart rates between species. Interestingly, as reported by Henson et al., end systolic LV torsion, once normalized to ventricular length, is comparable between healthy humans and mice and is argued to be a potential uniform measurement of normal ventricular ejection [33]. Average values were reported to be 2.7° in humans vs. 2.0° in mice, and 1.9°/cm in humans vs. 2.7°/cm in mice, for unnormalized and normalized torsion, respectively. For torsion rate, normalization of the rate of change of torsion throughout % systole rather than time is needed to compare values between humans and mice, whose torsion rate is approximately increased by a factor of four in the latter. In comparison to PAH in children, LV torsion rate in PAB mice demonstrated a steeper regressive relationship with RV contractility (Fig. 3b), and overall PAB mice had higher values of E_es_. This could perhaps be attributed to the difference in progression of the disease, in which PAB 7 weeks post-surgery represents an acute and more severe insult compared to the gradual worsening of PAH over time for human subjects. Furthermore, given that we are studying the pediatric population, age could have an impact on LV torsion, which slightly increases until adulthood [34]. Indeed, there is no ideal way to age-match an animal model to a clinical human population with drastically different life spans. However, these variations due to age are on the order of tenths of a degree [35] and fall well outside the derived confidence intervals for their respective groups. Drastic differences in heartrate between the two species (mice approximately 300–400 beats/min [35], children approximately 80–100 beats/min) could also be an influential factor accounting for this difference in LV torsion rate, given that it can have an impact on the computed end-systolic elastance [36]. Thus, with higher values of RV contractility, and through RV/PA coupling, PAB may represent a more progressed presentation of PAH and as such, the RV in PAB might be more sensitive to changes in LV torsion rate. This difference may be an indication that mechanical assistance from the LV is even more critical for the RV function in advanced stages of PAH. In progression of PAH, it is unknown whether RV dysfunction occurs prior to the reduction in LV torsion or vice versa. Longitudinal studies are needed to elucidate the mechanism of interventricular dependency in the development and progression of PAH and our PAB model thus might present a powerful tool to study this mechanism.

### Myocardial strain

Composite LV and RV strain parameters were comparable between PAB mice and PAH patients, and between Sham and control subjects. This demonstrates that studies interested in bi-ventricular mechanics in PAH can utilize the pre-clinical PAB mouse model as surrogate, despite such extreme differences in size and scale between the species. A recent study performed by Lapinskas et al. has demonstrated the validity and reproducibility of CMR-FT in small animals [37], and to our knowledge, we are the first to apply CMR-FT to a preclinical mouse model of PAH. It is intriguing that, although the mouse heart is approximately one thousand times smaller than an average child’s heart and beats roughly five times faster [35], the pressure experienced is similar between humans and mice (see Fig. 2b) [24]. Therefore, the measured similarity in myocardial strain between the two species—under roughly the same pressure—is likely explained by Laplace’s Law: WS = (P * r)/h, where WS is myocardial wall stress, P is pressure, r is ventricular radius, and h is wall thickness. Thus, given a similar pressure and consistent ratio of radius to wall thickness, myocardial wall stress and, hence, myocardial strain may theoretically be similar. However, this is a gross oversimplification of the ventricle as an idealized cylinder and assumes a thin wall, where the ratio of wall thickness to radius is less than one tenth. Interestingly, studies have shown 15–20% higher values of myocardial wall stress in humans compared to mice [36], thus, demonstrating intra-species differences in mechanical properties that may be due to higher myocardial stiffness within mice [38]. This phenomenon can be explained by same relationship as described by Laplace’s law [39, 40]. Because the integrity of the structure and shape of the ventricles is maintained between humans and mice, the ratio of LV radius to LV wall thickness is roughly equivalent and thus, with similar pressure, the resulting myocardial stress and strain is comparable between species. Indeed, our comparison below on various strain parameters between PAB and PAH patients is a reflection of this relationship.

LV global peak circumferential strain was reduced in both PAH patients and PAB mice compared to the control healthy/Sham groups. The decrease in LV circumferential strain is concomitant with a reduction in LV torsion rate. However, it is unknown whether one metric is secondary to the other. Combined, this data shows there is an overall decrease in LV circumferential motion and twisting capacity in the pressure overloaded RV. Increased pressure and volume within the RV causes a shift in the interventricular septum to flatten and encroach on the LV chamber, resulting in distortion of the shape of the LV and smaller LV volume [41, 42], thus, being a major contributor to LV mechanics and myocardial stress [43, 44]. Reduction in LV systolic strain has also been shown to be a predictor of poor outcome in adults with PAH [42]. RV global peak radial and longitudinal strain were reduced in both PAB and PAH patients. In contrast from the LV, the majority of RV contractility is generated by basal movement toward the apex through longitudinal strain [45, 46]. Thus, reduction in RV longitudinal strain would have a significant impact on RV contractility. To this end, previous research has reported reduced RV longitudinal strain as an independent predictor of RV failure [47]. A brief look at a correlation matrix with LV torsion rate and every independent strain parameter yielded a significant correlation with peak longitudinal RV displacement, which may be further evidence of LV torsional influence on RV function. Interestingly, LV global peak radial strain and RV global peak circumferential strain was reduced in PAB but not in PAH patients, compared to their control counterparts. This could be due to the PAB model being evaluated at seven weeks post-surgery, where prolonged exposure to RV pressure overload has allowed for more advanced RV remodeling, thus possibly representing a more aggressive and end-stage form of PAH compared to our pediatric subjects, whose RV–LV structural changes are less severe. Indeed, for all RV/LV global directional strains, PAH and control subjects demonstrated higher variance compared to that of their PAB and Sham counterparts. This is perhaps due to the heterogenous representation of the pediatric cohort, where there is a wide age range (11 years for both PAH/control) and patients are at different stages of disease, which could result in differences in ventricular remodeling and adaptation, and hence wider deviations in myocardial strain. From a mechanical perspective, we can see that prolonged RV pressure overload alone induces significant changes in both LV and RV structural mechanics in association with RV dysfunction.

### Animal model rationale

Various animal models exist for PAH research. However, there is no model that perfectly recapitulates the critical features of PAH in humans [48]. We chose the banding rodent model with constriction of the proximal PA as our experimental model to understand changes in myocardial mechanics in response to a purely acute mechanical insult without interfering with afterload alterations over time and without an insult to the pulmonary vasculature. Furthermore, while rat models are thought to be more robust rodent models of PAH [49], we chose a mouse model which can be combined with mouse genetic tools to specifically manipulate gene expression in a spatial and temporal manner [50].

Some have also argued that increased pressure alone does not fully induce right heart failure in PAH [26]. Monocrotaline (MCT), chronic hypoxia, and Sugen-hypoxia (Su-Hx) are well studied and commonly used models in PAH research [51, 52]. However, MCT also effects the liver and causes myocarditis in both ventricles, which may convolute the study of RV hypertrophy and fibrosis in PAH [51, 53]. In the hypoxic model, there are notable differences between species and variance with age, and also mainly represents the vasoconstrictive component with distal muscularization as a mild form of PAH, which is reversible and is not representative of PAH [54]. The Su-Hx model combines Sugen 5416, a vascular endothelial cell growth factor (VEGF) receptor inhibitor, with chronic hypoxia to induce a severe and irreversible form of PH [51]. However, studies of the RV and LV in this model have to be interpreted with caution given that VEGF receptor inhibition might likely alter the angiogenic capacity of the capillaries in the heart in response to RV hypertrophy and therefore worsen capillary rarefaction [53, 54]. The PAB model in particular offers the potential opportunity to study the molecular, structural and mechanical processes of both ventricles independent of the pulmonary vascular pathology.

## Limitations

Our study has several limitations. First, our chosen rodent model does not identically mimic human PAH as the pulmonary vasculature remains unaltered. However, as stated in the previous section, the PAB mouse is arguably a useful model for study of ventricular mechanics in the setting of acute mechanical insult. Second, we acknowledge that the mean age difference of the pediatric populations for PAH and control subjects could be a cause for concern in which the variation in duration of RV overload could result in a difference in RV remodeling and adaptation between individual subjects. Third, the possibility of higher variance within feature-tracking measurements on mice due to the inherent lower resolution of CMR images should be considered. However, our agreement studies validate our analyses and demonstrate that our experimental data is well outside our confidence intervals of both interobserver and intraobserver variance. Fourth, we did not consider longitudinal analysis or causality, as this was a pilot study in validating tagged-CMR and CMR-FT in our rodent model. Future studies will include CMR at multiple time points from time of surgery to seven weeks post-surgery to fully document the transformation of myocardial mechanics in progression of PAH. Our future research will also explore the causal effect of the LV–RV mechanical relationship and its influence on RV functional decline in development of PAH through alterations of contractile protein expression.

## Conclusion

Our pilot study demonstrates preliminary evidence that myocardial strain and torsion derived by tagged-CMR and CMR-FT in a rodent model of RV pressure overload may be a suitable model of ventricular mechanics in pediatric PAH. LV torsion rate and myocardial strain are reduced in PA-banded mice and in children with PAH and are associated with reduced RV contractility in both species. This study further highlights the critical role of interventricular mechanical dependency and its association with RV functional decline in PAH.

## Supplementary Information

**Additional file 1:** **Figure S1**. Intraobserver and Interobserver agreement for CMR-FT myocardial strains. Bland-Altman plots for (a) Intraobserver agreement of LV global peak circumferential strain (ICC, 0.92; mean difference, 0.853; 95% confidence interval − 0.10 to 1.8), and (b) Interobserver agreement of LV global peak circumferential strain (ICC, 0.85; mean difference, 1.08; 95% confidence interval − 0.12 to 2.28).

## Data Availability

The datasets used and/or analyzed during the current study are available from the corresponding author on reasonable request.

## Abbreviations

AR: Apical rotation
BR: Basal rotation
bSSFP: Balanced steady state free precession
CHD: Congenital heart disease
CMR: Cardiovascular magnetic resonance
CO: Cardiac output
E_es_: End-systolic elastance
E_a_: Arterial elastance
ECG: Electrocardiogram
FT: Feature tracking
LV: Left ventricle/left ventricular
LVEF: Left ventricular ejection fraction
MCT: Monocrotaline
mPAP: Mean pulmonary arterial pressure
PA: Pulmonary artery
PAB: Pulmonary arterial banding
PAH: Pulmonary arterial hypertension
PCA: Principle component analysis
PCWP: Pulmonary capillary wedge pressure
P_es_: End-systolic pressure
PP: Pulse pressure
PVR: Pulmonary vascular resistance
Rapex: Radius of apex
Rbase: Radius of base
RHC: Right heart catheterization
RV: Right ventricle/right ventricular
RVEF: Right ventricular ejection fraction
RVSP: Right ventricular systolic pressure
SPAMM: Spatial modulation of magnetization
Su-Hx: Sugen-hypoxia
SV: Stroke volume
T: Torsion
VEGF: Vascular endothelial cell growth factor
WHO: World Health Organization
WS: Wall stress
