# Supplementary material for: Pulmonary arterial banding in mice may be a suitable model for studies on ventricular mechanics in pediatric pulmonary arterial hypertension
Source: J Cardiovasc Magn Reson. 2021 Jun 3;23:66. doi: 10.1186/s12968-021-00759-8 (PMC8173855; doi:10.1186/s12968-021-00759-8)
Supplement: Supplementary file 1 — Additional file 1: Figure S1. Intraobserver and Interobserver agreement for CMR-FT myocardial strains. Bland-Altman plots for (a) Intraobserver agreement of LV global peak circumferential strain (ICC, 0.92; mean difference, 0.853; 95% confidence interval − 0.10 to 1.8), and (b) Interobserver agreement of LV global peak circumferential strain (ICC, 0.85; mean difference, 1.08; 95% confidence interval − 0.12 to 2.28). [file 12968_2021_759_MOESM1_ESM.pdf]

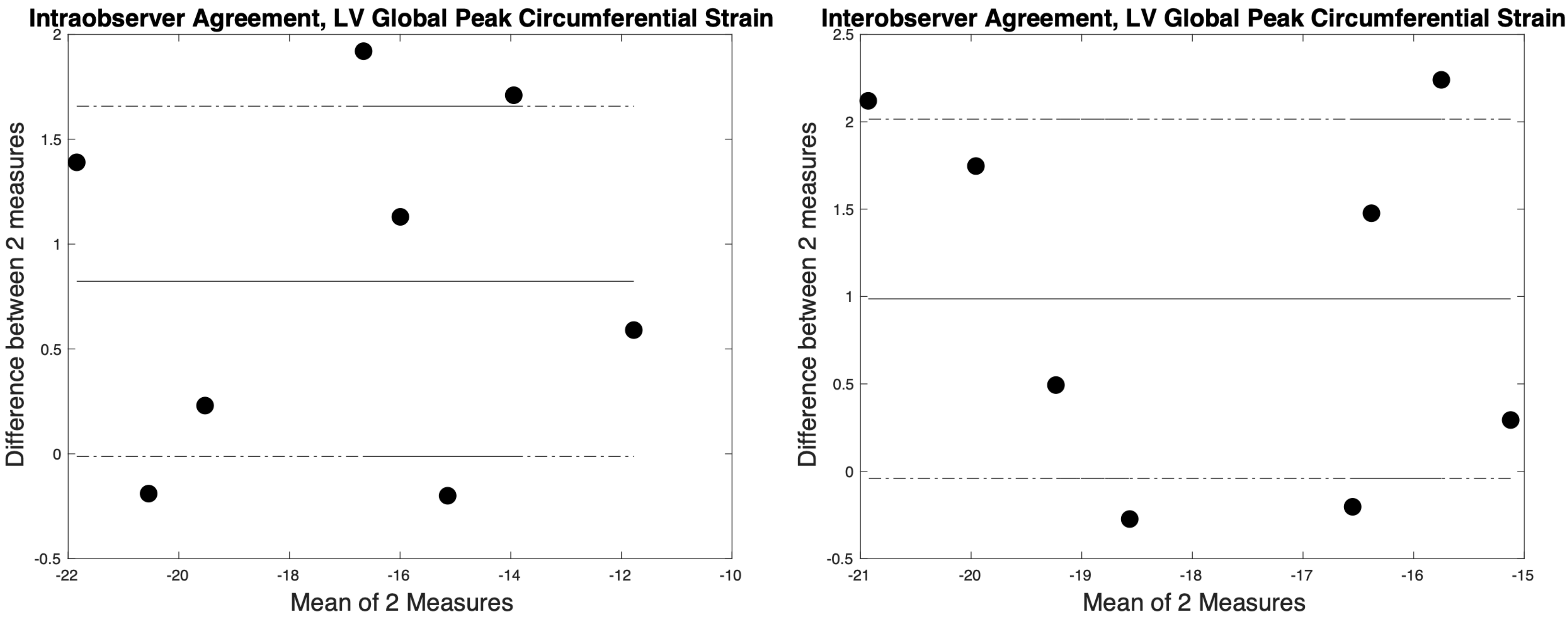

Intraobserver Agreement, LV Global Peak Circumferential Strain
Difference between 2 measures
Mean of 2 Measures
2
1.5
1
0.5
0
-0.5
-22
-20
-18
-16
-14
-12
-10
Interobserver Agreement, LV Global Peak Circumferential Strain
Difference between 2 measures
Mean of 2 Measures
2.5
2
1.5
1
0.5
0
-0.5
-21
-20
-19
-18
-17
-16
-15

a)

b)
